# Breed-Dependent Divergence in Breast Muscle Fatty Acid Composition Between White King and Tarim Pigeons

**DOI:** 10.3390/ani16010144

**Published:** 2026-01-05

**Authors:** Bo Zhang, Jiajia Liu, Hua Wei, Li Liu, Wanchao Zhang, Asmaa Taha Yaseen Kishawy, Li Shen, Jianyuan Ma, Yipu Li, Shuxian Xie, Haoxuan Li, Jing Li, Zheng Wang

**Affiliations:** 1 Institute of Animal Husbandry and Veterinary Medicine, Beijing Academy of Agriculture and Forestry Sciences, Beijing 100097, China; zhangb950414@163.com (B.Z.); 17320897356@163.com (L.S.); hpfpcj@163.com (J.M.); 18730978250@163.com (Y.L.); 17854271052@163.com (S.X.); crystalnihility@163.com (H.L.); xsu1980@163.com (J.L.); 2 Institute of Animal Husbandry, Xinjiang Uygur Autonomous Region Academy of Animal Science, Urumqi 830063, China; zbo62577@gmail.com; 3 The People’s Government of Moyu County, Moyu, Hotan 848199, China; xjhtxm@163.com; 4 Hotan Prefecture Animal Husbandry Technology Extension Station, Hotan 848099, China; xjliul@163.com (L.L.); htxmy0903@163.com (W.Z.); 5 Department of Nutrition and Clinical Nutrition, Faculty of Veterinary Medicine, Zagazig University, Zagazig 44519, Egypt; no.dispair2000@gmail.com

**Keywords:** Tarim pigeon, White King pigeon, fatty acid composition

## Abstract

Fatty acid composition of skeletal muscle is an important determinant of meat quality in poultry. In this study, we compared the breast muscle fatty acid profiles of White King (BW, n = 25) and Tarim (TM, n = 23) pigeons using targeted fatty acid quantification under identical feeding and management conditions. Sixteen differential fatty acids were identified, with TM pigeons exhibiting significantly higher levels of palmitic (C16:0), stearic (C18:0), oleic (C18:1n9c), and linoleic (C18:2n6c) acids (*p* < 0.001). Principal component analysis revealed clear separation between breeds, reflecting distinct fatty acid composition patterns. Kyoto Encyclopedia of Genes and Genomes annotation indicated that differential fatty acids were associated with pathways related to fatty acid biosynthesis, elongation, and degradation. Together, these results describe breed-specific differences in breast muscle fatty acid composition and provide a descriptive biochemical reference for understanding variation in meat quality-related traits among pigeon breeds.

## Simple Summary

Pigeons are an important poultry species used for meat production, and understanding their fatty acid composition is valuable for improving meat quality and supporting breeding research. In this study, we compared the breast muscle fatty acid profiles of two representative breeds—the high-yield White King pigeon and the local Tarim pigeon—raised under identical feeding and management conditions. Tarim pigeons showed higher levels of several fatty acids, including palmitic, stearic, oleic, and linoleic acids, whereas White King pigeons exhibited relatively lower overall fatty acid content. These results reflect intrinsic breed-associated differences in fatty acid composition and provide a reference for future studies on nutritional characteristics, genetic background, and breed evaluation of different pigeon populations.

## 1. Introduction

Pigeons (Columba livia) are valued for their high-quality meat and favorable nutritional attributes, a reputation historically reflected in the traditional Chinese saying “one pigeon is worth nine chickens” [1,2,3]. Among commercial breeds, the White King (BW) pigeon—developed through a four-way cross in the United States—has become the predominant meat-type pigeon in China owing to its rapid growth rate, large body size, and high meat yield [4,5]. In contrast, the Tarim (TM) pigeon is an indigenous Chinese breed originating from the Tarim Basin and Yarkand River region of Xinjiang. Shaped by long-term natural and artificial selection under arid desert conditions characterized by drought, large temperature fluctuations, and limited feed resources, TM pigeons exhibit strong environmental tolerance, stable production performance, and distinct morphological characteristics [6,7,8,9,10]. In addition to their production value, TM pigeons possess substantial cultural and historical importance and are recognized as a nationally protected genetic resource, representing an important component of local avian biodiversity.

Because BW and TM pigeons represent markedly different breeding histories—one shaped by intensive selection for rapid growth and muscle yield, and the other maintained through long-term local utilization and adaptation—comparisons between these two breeds provide a useful framework for examining how divergent selection backgrounds are associated with differences in muscle biochemical composition. Rather than assuming equivalent genetic or physiological states between breeds, such comparisons allow for a descriptive assessment of breed-related variation under standardized conditions.

Lipids are key constituents of skeletal muscle and play important roles in determining meat quality attributes such as flavor, tenderness, and nutritional value [11,12]. In poultry species such as chickens and ducks, fatty acid profiling has been widely used to characterize breed differences and evaluate meat quality-related traits [13,14]. However, systematic analyses of muscle fatty acid composition in pigeons—particularly in indigenous and culturally significant local breeds—remain limited.

In the present study, we conducted targeted fatty acid quantification in the breast muscle of BW and TM pigeons raised under identical feeding and management conditions. By characterizing breed-specific differences in fatty acid composition, this study provides a descriptive biochemical reference for understanding variation in muscle lipid characteristics between commercial and local pigeon breeds and offers baseline data to support future research on pigeon meat quality and genetic resource conservation.

## 2. Materials and Methods

The experimental procedures were approved by the Animal Welfare Committee of the Institute of Animal Husbandry and Veterinary Medicine, Beijing Academy of Agriculture and Forestry Sciences (Approval NO. IHVM11-2304-20).

### 2.1. Animals

The BW and TM pigeons used in this study were obtained from Jiangsu Weitekai Pigeon Industry Co., Ltd. (Jiangyin, China) A total of 200 healthy breeding pairs of each breed with similar body weight and size were selected. All pigeons were managed under standardized housing and feeding conditions in accordance with the Chinese National Standard GB/T 36196-2018 (Standardization Administration of China, Beijing, China, 2018) “Technical regulation of feeding and management for egg pigeon” to ensure consistent environmental parameters.

The pigeons were housed in double-deck cages (one breeding pair per cage, 50 cm length × 50 cm deep × 60 cm height) and were provided free access to a corn–soybean–sorghum–wheat grain-based diet, supplemented with a healthy vitamin–mineral–sand mixture, also consistent with GB/T 36196–2018 recommendations. Although the fatty acid profile of the diet was not chemically analyzed, both breeds consumed the same diet throughout the experiment, eliminating dietary differences between groups. The main ingredients of the raw grains included corn (approximately 40%), beans (22%), sorghum (19%), wheat (19%), and a vitamin–mineral premix. The estimated metabolizable energy of the diet was 12.5 MJ/kg, and the crude protein content was 15.9%. The crude fat content in the raw grains was determined to be 2.85% (by the Soxhlet extraction method, according to GB/T 6433-2025 (Determination of crude fat in feeds, State Administration for Market Regulation & Standardization Administration of China, Beijing, China, 2025).

Environmental conditions (temperature, humidity, ventilation, and illumination) were maintained within the ranges specified in egg pigeon production. Specifically, the ambient temperature was kept at 18 °C~24 °C and relative humidity at 75%, in accordance with the Chinese Agricultural Industry Standard (NY/T 388-1999, Environmental quality standard for the livestock and poultry farm, Ministry of Agriculture of the People’s Republic of China, Beijing, China, 1999). Artificial light was provided from 17:00 daily to ensure a 16 h photoperiod.

To minimize potential confounding effects and ensure the biological independence of samples, only male squabs were used for fatty acid analysis. A total of 25 BW and 23 TM male squabs were collected at 28 days of age. Importantly, each sampled squab originated from a different breeding pair and a different nest, meaning that no two individuals were derived from the same parents. Therefore, each squab constituted an independent biological replicate. Although 200 breeding pairs per breed were maintained, several practical constraints influenced the final number of available samples. Pigeons lay only two eggs per clutch, and laying cannot be synchronized across large flocks, resulting in a prolonged sampling period. In addition, a proportion of the offspring had been used for carcass trait measurements in our previous study [10]. These carcass samples underwent scalding and defeathering, which made them unsuitable for fatty acid profiling. Consequently, the samples used in the present study were obtained from the remaining offspring, specifically collected for lipid analysis. All selected squabs were raised under identical feeding, housing, and management conditions to ensure consistency of environmental factors.

### 2.2. Slaughter and Sample Collection

At the end of the experiment, pigeons were euthanized by gradual-fill CO_2_ inhalation in accordance with institutional animal care guidelines. CO_2_ was introduced at a controlled displacement rate until loss of consciousness, and euthanasia was confirmed by the absence of respiration and cardiac activity. Slaughtering and sampling were conducted between 09:00 and 11:00 to reduce circadian variation. Immediately after exsanguination, the left breast muscle was excised within 5 min post-mortem, placed into 1.5 mL EP tubes, snap-frozen in liquid nitrogen, and stored at −80 °C until analysis.

### 2.3. Lipidomic Analysis

#### 2.3.1. Metabolite Extraction

Biological samples were first lyophilized and ground into a fine powder using a homogenizer. Approximately 100 mg of the powder was extracted with 1.2 mL of 70% methanol. The mixture was vortexed for 30 s every 30 min, for a total of six times, and then incubated at 4 °C overnight. Following centrifugation, the supernatant was collected, filtered through a microporous membrane, and transferred into sample vials for UPLC-MS/MS analysis.

#### 2.3.2. UPLC-MS/MS Conditions

Chromatographic separation was performed on an Agilent SB-C18 column (1.8 μm, 2.1 × 100 mm). The mobile phases consisted of 0.1% formic acid in water (A) and 0.1% formic acid in acetonitrile (B). The flow rate was 0.35 mL/min, the injection volume was 4 μL, and the column temperature was maintained at 40 °C. Mass spectrometric detection was conducted using a QTRAP LC–MS/MS system (Sciex) operating in both positive and negative ion modes.

#### 2.3.3. LC-MS/MS Analysis

LIT and triple quadrupole (QQQ) data were obtained using a QTRAP LC-MS/MS system (Sciex, Framingham, MA, USA) equipped with an electrospray ionization Turbo Ion-Spray source, operating in both positive and negative ion modes, and controlled by Analyst software (version 1.6.3). The ESI source parameters were set as follows: source temperature, 500 °C; ion spray voltage, +5500 V (positive) and −4500 V (negative); ion source gas I (GS I) and gas II (GS II); curtain gas pressures were 55, 60, and 25 psi, respectively; and collision gas was maintained at a high setting. Mass calibration and instrument tuning were conducted using 10 and 100 μmol/L polypropylene glycol solutions for QQQ and LIT modes, respectively. Multiple reaction monitoring (MRM) transitions were optimized and monitored according to the retention time of the eluting metabolites.

#### 2.3.4. Quality Control (QC) Strategy

A pooled QC sample was prepared by mixing equal aliquots of all study samples. QC samples were injected after every 10 analytical samples to monitor signal stability. QC reproducibility was evaluated using principal component analysis (PCA) clustering and the coefficient of variation (CV) of internal features (<20%). This ensured instrument stability and analytical reliability throughout the analysis.

### 2.4. Enzyme Activity and Muscle Glycogen Measurements

To further characterize biochemical properties of the breast muscle, the activities of key metabolic enzymes and muscle glycogen content were measured using commercial assay kits. Carnitine palmitoyltransferase 1 (CPT1) activity was determined using a CPT1 assay kit (Bioss, Beijing, China; catalog no. AK491V). Muscle glycogen content was measured using a glycogen assay kit (Nanjing Jiancheng Bioengineering Institute, Nanjing, China; catalog no. A043-1-1). Citrate synthase (CS) activity was analyzed using a citrate synthase assay kit (Solarbio Science & Technology Co., Ltd., Beijing, China; catalog no. BC1060). Lactate dehydrogenase (LDH) activity was determined using a lactate dehydrogenase assay kit (Nanjing Jiancheng Bioengineering Institute, Nanjing, China; catalog no. A020-2-2).

All assays were performed strictly according to the manufacturer’s instructions. Enzyme activities and glycogen concentrations were calculated following the provided protocols and normalized to protein content or tissue weight, as specified by each assay kit.

### 2.5. Data Processing and Statistical Analysis

Raw peak areas were normalized to total ion current (TIC). For the targeted fatty acid panel, quantification was based on multiple reaction monitoring (MRM) transitions. Although internal standards were not used in this study, QC samples and TIC normalization were employed to minimize analytical bias. PCA was used to evaluate sample clustering. OPLS-DA models were constructed using the ropls R package (version 4.3.1), and 200-time permutation tests were used to assess model reliability. Fold change (FC) and Student’s *t*-test were used to identify differences between breeds. Data normality was tested using the Shapiro–Wilk test, and homogeneity of variances was assessed using Levene’s test. For both tests, *p*-values greater than 0.05 were considered to meet the assumptions required for parametric statistical analysis. For all variables, Shapiro–Wilk test *p*-values ranged from 0.12 to 0.89, and Levene’s test *p*-values ranged from 0.21 to 0.77, indicating that the assumptions of normality and homogeneity of variances were satisfied. Differential fatty acids were defined by *FC* > 1.5 and *p* < 0.05. KEGG enrichment was performed using a hypergeometric distribution test.

## 3. Results

### 3.1. Comparison of Fatty Acid Composition Between BW and TM Pigeons

**Figure 1:**
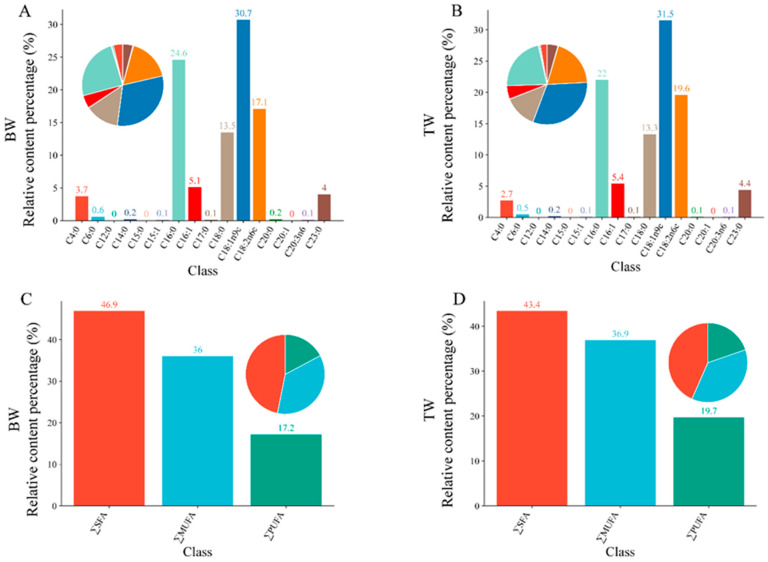
Fatty acid composition of breast muscles in White King (BW) and Tarim (TM) pigeons. (**A**,**B**) Relative contents (%) of individual fatty acids in the breast muscles of BW (**A**) and TM (**B**) pigeons. (**C**,**D**) Total proportions of saturated fatty acids (ΣSFA), monounsaturated fatty acids (ΣMUFA), and polyunsaturated fatty acids (ΣPUFA) in BW (**C**) and TM (**D**) pigeons. Values represent mean percentages of total identified fatty acids. The pie charts indicate the proportional distribution of different fatty acid classes within each breed.

Targeted lipidomics identified 16 fatty acids in the breast muscles of BW and TM pigeons (Figure 1; Table 1, Supplement Figure S1). Among these, several fatty acids showed substantial contributions to breed differences based on three criteria: high abundance, significant fold-changes, and high multivariate importance (*VIP* scores).

According to Table 1 and Supplement Figure S1, palmitic acid (C16:0), stearic acid (C18:0), oleic acid (C18:1n9c), and linoleic acid (C18:2n6c) were the major fatty acids in pigeon breast muscle, representing the principal components of SFA, MUFA, and PUFA pools. These fatty acids also showed the most pronounced quantitative differences between breeds. Compared with BW pigeons, TM pigeons exhibited markedly higher levels of C16:0, C18:0, and C18:1n9c, with fold increases of 1.79-, 1.97-, and 2.05-fold, respectively (*p* < 0.001). These high-abundance fatty acids also ranked among the top contributors in the OPLS-DA model, showing *VIP* scores of 1.85 (C16:0), 1.51 (C18:0), and 2.81 (C18:1n9c), indicating strong discriminatory power between breeds (Table 2).

At the class level, TM pigeons exhibited significantly higher total SFA, MUFA, and PUFA contents: 1.85-, 2.06-, and 2.29-fold greater than BW pigeons, respectively (all *p* < 0.001; Figure 1C,D). Despite these increases, the ratios of ΣMUFAs/ΣSFAs and ΣPUFAs/ΣMUFAs remained statistically unchanged (*p* > 0.05).

### 3.2. Comparative Fatty Acid Profiling Between BW and TM Pigeons

**Figure 2:**
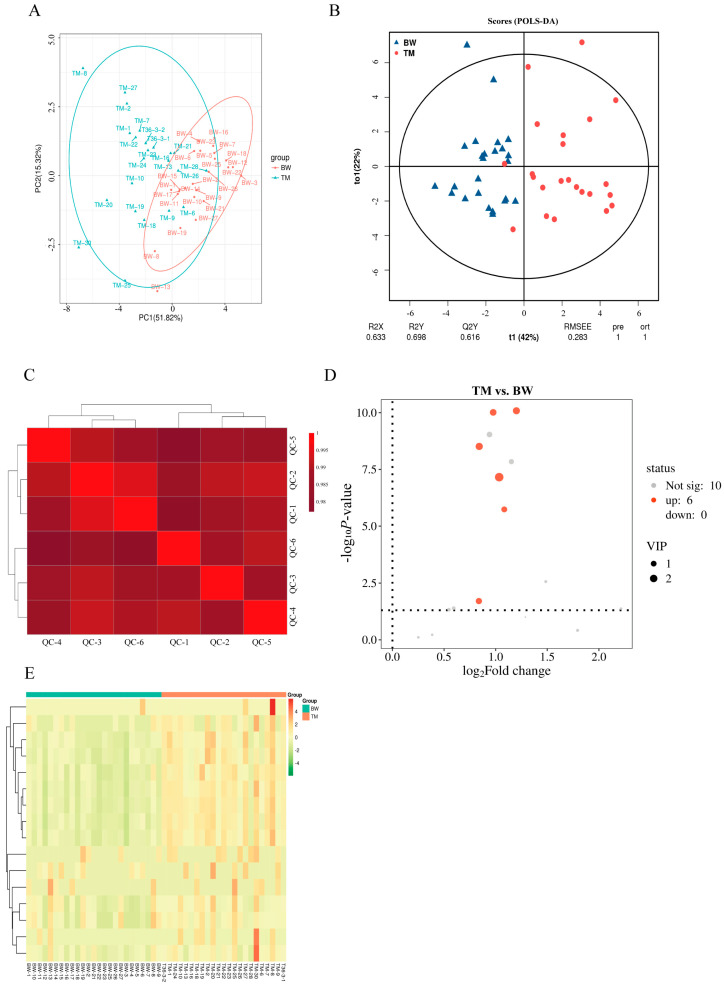
Multivariate and differential fatty acids profile analyses of lipid metabolites in the breast muscle of White King (BW) and Tarim (TM) pigeons. (**A**) Principal component analysis (PCA) showing clear separation between BW and TM groups based on lipid metabolite profiles. (**B**) Orthogonal partial least squares–discriminant analysis (OPLS-DA) demonstrating strong model predictability (R^2^X = 0.693; R^2^Y = 0.689; Q^2^ = 0.616). (**C**) Quality control (QC) sample correlation heatmap showing high consistency among QC injections (Pearson correlation coefficients > 0.98). (**D**) Volcano plot illustrating significantly altered metabolites between breeds (red: upregulated in TM; black: unchanged). Key differential fatty acids are labeled, including palmitic acid, stearic acid, linoleic acid, myristic acid, and tricosanoic acid. (**E**) Hierarchical clustering heatmap showing distinct metabolite expression patterns between BW and TM groups. Each point represents an independent biological replicate (BWn = 25 and TMn = 23). *VIP* > 1.0 and *p* < 0.05 were used as screening thresholds for significant differential metabolites.

To further characterize breed-dependent differences in muscle lipid composition, targeted fatty acid profiling was performed in the pectoral muscles of BW and TM pigeons (Figure 2). Principal component analysis (PCA) revealed a clear separation between the two breeds based on fatty acid composition, indicating distinct breed-associated fatty acid profiles (Figure 2A). This separation was further supported by orthogonal partial least squares–discriminant analysis (OPLS-DA), which showed good model fitness and predictive performance (R^2^X = 0.693; R^2^Y = 0.689; Q^2^ = 0.616; Figure 2B).

Quality control (QC) clustering analysis demonstrated high analytical reproducibility and stability of the fatty acid measurements (Figure 2C). Volcano plot analysis identified six fatty acids that differed significantly between BW and TM pigeons (*VIP* > 1, *p* < 0.05; Figure 2D). Hierarchical clustering analysis based on these differential fatty acids revealed distinct fatty acid composition patterns between the two breeds, further supporting the multivariate analysis results (Figure 2E).

### 3.3. Breed-Dependent Differences in Fatty Acid Composition

Based on the identified lipid metabolites, fatty acids were grouped into three categories: saturated fatty acids (ΣSFA), monounsaturated fatty acids (ΣMUFA), and polyunsaturated fatty acids (ΣPUFA). As shown in Figure 1 and Table 1, the relative proportions of all three categories differed significantly between breeds. TM pigeons exhibited higher contents of ΣSFA (46.9%), ΣMUFA (36.0%), and ΣPUFA (17.2%) compared with BW pigeons (43.4%, 34.9%, and 19.7%, respectively), indicating enhanced overall lipid deposition in the TM breed.

Further compositional analysis demonstrated that C14:0, C16:0, C16:1, C17:0, C18:0, C18:1n9c, C18:2n6c, and C23:0 were the main contributors to these differences, with TM pigeons showing significantly higher levels of these fatty acids (*p* < 0.05; Table 1).

### 3.4. Kyoto Encyclopedia of Genes and Genomes (KEGG) Pathway Enrichment Analysis of Differential Lipid Metabolites

**Figure 3:**
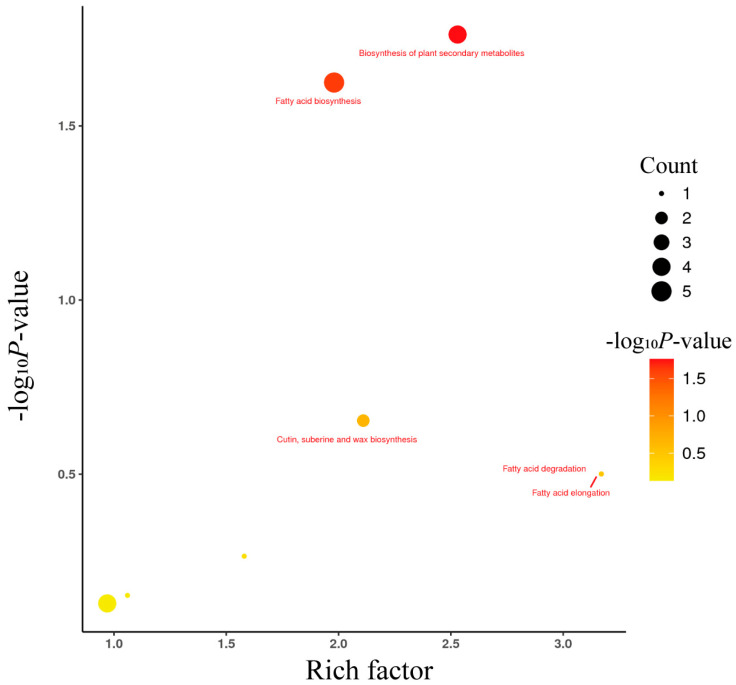
KEGG pathway enrichment analysis of differential lipid metabolites between White King (BW) and Tarim (TM) pigeons.

KEGG pathway enrichment analysis revealed that differential lipid metabolites between BW and TM pigeons were mainly involved in fatty acid biosynthesis, fatty acid elongation, fatty acid degradation, cutin, suberine and wax biosynthesis, and the biosynthesis of secondary metabolites (Figure 3). Among these, the fatty acid biosynthesis (*p* = 0.0173) and biosynthesis of secondary metabolites (*p* = 0.0237) pathways exhibited the highest enrichment significance.

### 3.5. Comparison of Muscle Metabolic Enzyme Activities Between BW and TM Pigeons

To further characterize metabolic differences associated with muscle lipid and energy metabolism, the activities of key metabolic enzymes and muscle glycogen content were measured in the breast muscles of BW and TM pigeons (Table 3).

As shown in Table 3, TM pigeons exhibited significantly higher activities of CPT1 and citrate synthase compared with BW pigeons (*p* < 0.001). Specifically, CPT1 activity in TM pigeons was 9.37 nmol/min·mg protein, which was approximately 2.6-fold higher than that observed in BW pigeons (3.60 nmol/min·mg protein). Similarly, CS activity was markedly elevated in TM pigeons (17.9 U/mg protein) relative to BW pigeons (2.94 U/mg protein, *p* < 0.001).

In addition, muscle glycogen content was significantly higher in TM pigeons than in BW pigeons (0.225 vs. 0.185 mg/g, *p* < 0.001). In contrast, LDH activity was significantly greater in BW pigeons (12.8 U/g protein) than in TM pigeons (6.70 U/g protein, *p* < 0.001).

Bubble plot illustrating the top enriched KEGG pathways identified from differential lipid metabolites (*VIP* > 1, *p* < 0.05). The *x*-axis represents the rich factor, and the *y*-axis shows the log_10_ (*p* value), indicating enrichment significance. Bubble size corresponds to the number of annotated metabolites within each pathway, while the color gradient from yellow to red reflects increasing significance levels. The most significantly enriched pathways include fatty acid biosynthesis, fatty acid elongation, fatty acid degradation, cutin, suberine and wax biosynthesis, and biosynthesis of plant secondary metabolites.

## 4. Discussion

In this study, we compared the breast muscle fatty acid composition of BW and TM pigeons raised under identical feeding conditions, housing, and management. Importantly, all sampled individuals were males, which reduced variability associated with sex-dependent differences in lipid metabolism. Therefore, the observed differences primarily reflect intrinsic breed variation.

### 4.1. Breed-Associated Differences in Fatty Acid Composition

TM pigeons exhibited higher levels of saturated (ΣSFAs), monounsaturated (ΣMUFAs), and polyunsaturated fatty acids (ΣPUFAs) compared with BW pigeons. In particular, palmitic acid (C16:0), stearic acid (C18:0), oleic acid (C18:1n9c), and linoleic acid (C18:2n6c) were markedly enriched in TM pigeons (Figure 1, Table 1), consistent with previous studies showing that higher SFA and MUFA content correlates with greater lipid deposition efficiency in avian muscle [15,16]. Conversely, BW pigeons exhibited relatively lower lipid content, consistent with a metabolic profile favoring lean muscle development [17,18]. These differences reflect inherent breed-specific patterns of fatty acid accumulation.

Importantly, these results should be interpreted as differences in fatty acid composition rather than direct evidence of divergent metabolic strategies.

### 4.2. Key Fatty Acids and Potential Physiological Relevance

Several individual fatty acids, including C14:0, C16:0, C16:1, C17:0, C18:0, C18:1n9c, C18:2n6c, and C23:0, were consistently higher in TM pigeons (*p* < 0.05; Table 1). The observed fold changes (approximately 1.5–2.5×) fall within ranges commonly reported as biologically relevant for intramuscular lipid deposition in poultry species [15,16,19,20].

Previous studies have shown that variations in the relative abundance of these fatty acids can influence membrane composition, lipid storage capacity, and oxidative stability of muscle tissue [20,21,22]. In the present study, however, these associations are interpreted at the compositional level only, as no direct measurements of lipid turnover, metabolic flux, or mitochondrial function were conducted.

### 4.3. Muscle Metabolic Enzyme Activities and Glycogen Content

To complement the lipidomic data, we measured the activity of selected metabolic enzymes and muscle glycogen content (Table 3). TM pigeons exhibited significantly higher activities of CPT1 and CS compared with BW pigeons (*p* < 0.001), while BW pigeons showed higher LDH activity. In addition, muscle glycogen content was modest but higher in TM pigeons.

These measurements provide limited but direct biochemical evidence of differences in muscle metabolic characteristics between breeds. However, they do not allow comprehensive conclusions regarding overall energy utilization patterns or preferential metabolic pathways. Rather, they suggest that breed-associated differences in fatty acid composition are accompanied by measurable variation in selected metabolic indicators under standardized conditions.

### 4.4. Implications for Pigeon Breeding, Local Breed Conservation, and Future Research

The breed-dependent differences in fatty acid composition observed in this study, including elevated levels of palmitic acid (C16:0), stearic acid (C18:0), oleic acid (C18:1n9c), and linoleic acid (C18:2n6c) in TM pigeons, reflect inherent biochemical characteristics associated with breed identity under standardized rearing conditions. These differences provide insight into variation in muscle lipid composition and are consistent with previous reports linking fatty acid profiles to meat quality attributes and nutritional value in poultry species [23,24].

From a breeding and resource management perspective, the contrasting fatty acid profiles between the intensively selected BW pigeons and the locally maintained TM pigeons highlight the biochemical consequences of long-term selection histories. Rather than implying adaptive superiority, these differences may serve as descriptive biochemical markers that contribute to breed characterization and evaluation [25,26,27,28]. Such markers can complement traditional phenotypic and genetic assessments by providing an additional layer of information on muscle composition.

Furthermore, the fatty acid composition data generated in this study offer a reference framework for future investigations aimed at integrating lipid profiles with genetic, nutritional, and physiological traits. By combining fatty acid profiling with broader phenotypic and genomic datasets in subsequent studies, it may be possible to better understand the regulatory factors influencing lipid deposition and muscle characteristics in pigeons [29,30]. These findings therefore support the continued documentation and utilization of breed-specific traits while avoiding overinterpretation beyond the experimental evidence presented here.

### 4.5. Study Limitations

While this study provides a systematic comparison of overall fatty acid composition in the breast muscle of BW and TM pigeons under controlled feeding and housing conditions, several limitations should be acknowledged.

First, the analytical scope was limited to a targeted set of fatty acids, rather than a comprehensive characterization of lipid classes or broader metabolic profiles. Therefore, variation in specific lipid species or pathways beyond overall fatty acid composition was not evaluated, and the results should be interpreted strictly at the level of fatty acid abundance. Second, the study focused on a single tissue (breast muscle) and a single developmental stage, which does not capture potential temporal or tissue-specific variation in fatty acid deposition. Finally, no direct measurements of carbohydrate-related parameters, mitochondrial activity, or metabolic flux were conducted. Accordingly, the present data do not support inferences regarding energy utilization, metabolic flexibility, or physiological adaptation, and interpretations are confined to descriptive differences in fatty acid composition between breeds.

Despite these limitations, the findings offer a well-controlled reference for breed-associated variation in muscle fatty acid composition and provide a basis for future studies that may integrate fatty acid data with physiological, molecular, or genetic analyses.

## 5. Conclusions

Taken together, this study demonstrates clear breed-associated differences in breast muscle fatty acid composition between TM and BW pigeons reared under standardized feeding and management conditions. TM pigeons exhibited higher overall abundance of multiple fatty acids, whereas BW pigeons displayed comparatively lower fatty acid levels, reflecting intrinsic breed-specific biochemical characteristics rather than evidence of divergent metabolic strategies.

Several limitations should be acknowledged. The present analysis was restricted to a targeted panel of fatty acids, and other lipid classes, metabolites, and molecular pathways were not examined. In addition, transcriptomic and proteomic data were not included, and the comparison was conducted at a single developmental stage under controlled environmental conditions. Accordingly, the findings should be interpreted as descriptive differences in fatty acid composition rather than indicators of metabolic regulation or physiological adaptation.

Future studies incorporating broader lipid class profiling, transcriptomic and proteomic analyses, and multiple breeds or developmental stages, together with controlled nutritional or environmental interventions, will be necessary to strengthen causal inference and to further elucidate the regulatory mechanisms underlying fatty acid deposition and muscle development in pigeons.

## Figures and Tables

**Table 1:** Fatty acid composition of breast muscles in BW and TM pigeons.

Name	Abbreviation	BW	TM	Fold Change	*p*-Value
Butyric acid	C4:0	5.15	7.53	1.46	0.0468
Hexanoic acid	C6:0	0.865	1.31	1.51	0.0407
Lauric acid	C12:0	0.0188	0.0458	2.44	0.0996
Myristic acid	C14:0	0.326	0.626	1.92	9.19 × 10^−10^
Pentadecanoic acid	C15:0	0.0124	0.0575	4.64	0.0425
cis-10-Pentadecenoic acid	C15:1	0.154	0.183	1.19	0.777
Palmitic acid	C16:0	34.0	60.8	1.79	3.07 × 10^−9^
Palmitoleic acid	C16:1	7.04	14.9	2.12	1.83 × 10^−6^
Heptadecanoic acid	C17:0	0.0940	0.263	2.80	0.00272
Stearic acid	C18:0	18.6	36.6	1.97	9.71 × 10^−11^
Oleic acid	C18:1n9c	42.5	87.0	2.05	6.95 × 10^−8^
Linoleic acid	C18:2n6c	23.6	54.2	2.30	8.23 × 10^−11^
Arachidic acid	C20:0	0.213	0.381	1.79	0.0196
11-Eicosenoic acid	C20:1	0.0157	0.0545	3.47	0.384
cis-8,11,14-Eicosatrienoic acid	C20:3n6	0.163	0.213	1.31	0.599
Tricosanoic acid	C23:0	5.46	12.1	2.22	1.44 × 10^−8^
∑SFA		64.7	120	1.85	<0.001
∑MUFA		49.7	102	2.06	<0.001
∑PUFA		23.7	54.4	2.29	<0.001
∑MUFA/∑SFA		0.783	0.846	1.08	0.257
∑PUFA/∑MUFA		0.494	0.553	1.12	0.170

Abbreviation: SFA, saturated fatty acids; MUFA, monounsaturated fatty acids; PUFA, polyunsaturated fatty acids. Sample size: 25 BW pigeons and 23TM pigeons.

**Table 2:** *VIP* scores of differential fatty acids.

Name	Abbreviation	*VIP* Score
Butyric acid	C4:0	0.235
Hexanoic acid	C6:0	0.284
Lauric acid	C12:0	0.0151
Myristic acid	C14:0	0.962
Pentadecanoic acid	C15:0	0.0857
cis-10-Pentadecenoic acid	C15:1	0.0661
Palmitic acid	C16:0	1.85
Palmitoleic acid	C16:1	1.07
Heptadecanoic acid	C17:0	0.115
Stearic acid	C18:0	1.51
Oleic acid	C18:1n9c	2.81
Linoleic acid	C18:2n6c	1.75
Arachidic acid	C20:0	1.26
11-Eicosenoic acid	C20:1	0.127
cis-8,11,14-Eicosatrienoic acid	C20:3n6	0.0599
Tricosanoic acid	C23:0	0.741

**Table 3:** The activity of the enzyme in the breast muscles of pigeons.

Item	BW	TM	SEM	*p*-Value
CPT1 (nmol/min·mgprot)	3.60 b	9.37 a	0.872	<0.001
Muscle glycogen (mg/g)	0.185 b	0.225 a	0.0062	<0.001
CS (U/mgprot)	2.94 b	17.9 a	2.28	<0.001
LDH (U/gprot)	12.8 a	6.70 b	0.955	<0.001

a, b represent different letters indicating significant differences between varieties, *p* < 0.05 (n = 6). Abbreviation: CPT1, carnitine palmitoyltransferase 1; CS, Citrate synthase; LDH, L-lactic dehydrogenase.

## Data Availability

Data are contained within the article.

## Supplementary Materials

The following supporting information can be downloaded at: https://www.mdpi.com/article/10.3390/ani16010144/s1, Figure S1: Classification and comparative analysis of differential fatty acids in the breast muscle of White King (BW) and Tarim (TM) pigeons.
