# Supplementary material for: Breed-Dependent Divergence in Breast Muscle Fatty Acid Composition Between White King and Tarim Pigeons
Source: Animals (Basel). 2026 Jan 5;16(1):144. doi: 10.3390/ani16010144 (PMC12784989; doi:10.3390/ani16010144)
Supplement: Supplementary file 1 [file animals-16-00144-s001.zip › animals-4003552-supplementary.pdf]

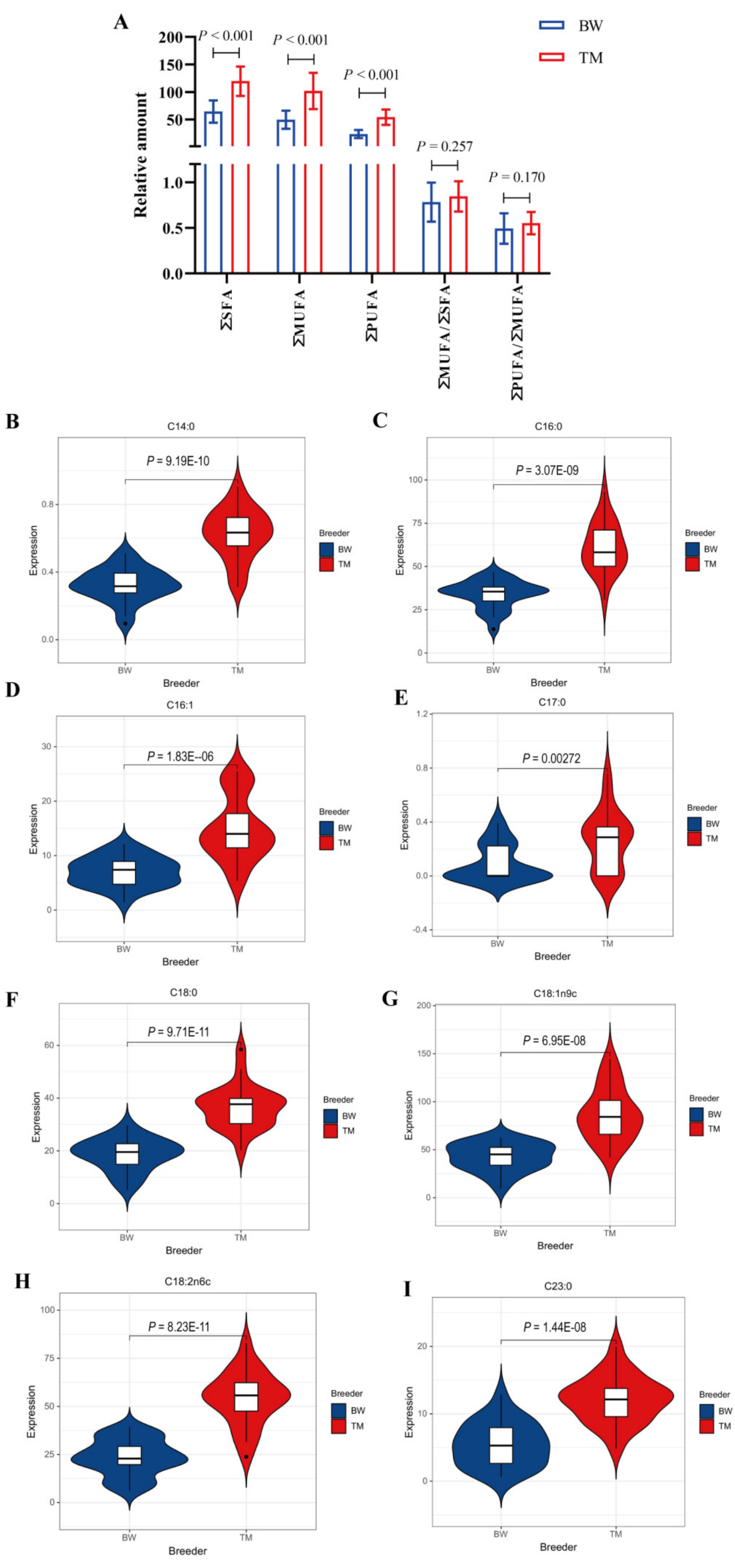
A
BW
TM
Relative amount
P < 0.001
P < 0.001
P < 0.001
P = 0.257
P = 0.170
ΣSFA
ΣMUFA
ΣPUFA
ΣMUFA/ΣSFA
ΣPUFA/ΣMUFA
B
C14:0
P = 9.19E-10
C
C16:0
P = 3.07E-09
D
C16:1
P = 1.83E--06
E
C17:0
P = 0.00272
F
C18:0
P = 9.71E-11
G
C18:1n9c
P = 6.95E-08
H
C18:2n6c
P = 8.23E-11
I
C23:0
P = 1.44E-08
Expression
Breeder
BW
TM

Supplement Figure 1. Classification and comparative analysis of differential fatty acids in the breast muscle of White King (BW) and Tarim (TM) pigeons.

(A) Relative composition of saturated (ΣSFA), monounsaturated (ΣMUFA), and polyunsaturated fatty acids (ΣPUFA) between breeds.

(B–I) Relative abundances of representative fatty acids that significantly differed between BW and TM pigeons, including C14:0, C16:0, C16:1, C17:0, C18:0, C18:1n9c, C18:2n6c, and C23:0.

Bars and violin plots represent mean ± SEM (BWn = 25 and TMn = 23). Statistical significance was determined by Student's t-test ($P < 0.05$).

Abbreviations: SFA, saturated fatty acids; MUFA, monounsaturated fatty acids; PUFA, polyunsaturated fatty acids.
